# Cardiovascular Disease in Post-Acute COVID-19 Syndrome: A Comprehensive Review of Pathophysiology and Diagnosis Approach

**DOI:** 10.31083/j.rcm2401028

**Published:** 2023-01-13

**Authors:** Nuraini Yasmin Kusumawardhani, Iwan Cahyo Santosa Putra, William Kamarullah, Rien Afrianti, Miftah Pramudyo, Mohammad Iqbal, Hawani Sasmaya Prameswari, Chaerul Achmad, Badai Bhatara Tiksnadi, Mohammad Rizki Akbar

**Affiliations:** ^1^Department of Cardiology and Vascular Medicine, Faculty of Medicine, University of Padjadjaran, 40132 Bandung, Indonesia; ^2^Department of Internal Medicine, Faculty of Medicine, University of Padjadjaran, 40132 Bandung, Indonesia; ^3^Emergency Department, R. Syamsudin SH Regional Public Hospital, Sukabumi, 43341 West Java, Indonesia

**Keywords:** COVID-19, long COVID-19, SARS-CoV-2, cardiovascular system, post-acute COVID-19, PACS

## Abstract

Long COVID or post-acute Coronavirus disease 2019 (COVID-19), a malady defined by the persistence of COVID-19 symptoms for weeks or even months, is expected to affect the lives of millions of individuals worldwide significantly. Cardiopulmonary symptoms such as chest discomfort, shortness of breath, fatigue, and autonomic manifestations such as postural orthostatic tachycardia syndrome, and arrhythmias are prevalent and widely recognized. A variety of cardiovascular problems, including myocardial inflammation, myocardial infarction, ventricular dysfunction, and endothelial dysfunction, have been described in individuals following the initial acute phase. With over 10,000 published publications on COVID-19 and the cardiovascular system, presenting an unbiased thorough analysis of how SARS-CoV-2 affects the system is essentially challenging. This review will provide an overview of frequent cardiovascular manifestations, emphasizing consequences, proposed pathophysiology, and clinical diagnostic manifestation strategy.

## 1. Introduction

The year 2020 was a momentous occasion in both history and global health. The Coronavirus disease 2019 (COVID-19) pandemic has emphasized the dangers of fatal epidemic-prone illnesses wreaking havoc on the globalized world. In Wuhan, China, pneumonia with anunknown origin became common in December 2019. RNA was isolated and sequenced from bronchoalveolar lavage fluid samples from these individuals. The culprit responsible for COVID-19 was discovered to be a new beta coronavirus, SARS-CoV-2, which has caused morbidity and mortality on an unparalleled worldwide scale [1, 2]. The COVID-19 pandemic has been ongoing for more than two years, with no end in sight in the near future. A significant number of organ dysfunctions have been discovered as a result of considerable and ongoing studies on COVID-19.

While the pharmaceutical armamentarium for COVID-19 is still being developed in order to minimize morbidity and death in COVID-19 patients, health communities must contend with a unique condition experienced by some COVID-19 survivors. This syndrome is associated with persistent symptoms and/or delayed or long-term complications beyond four weeks from the onset of symptoms, known as long haulers, long COVID, or post-acute COVID-19 syndrome (PACS) [3].

Some of the symptoms and signs observed in long-term COVID-19 patients relate to cardiovascular problems, accounting for roughly 42% of PACS symptoms. Furthermore, laboratory data and imaging reveal cardiovascular problems in long COVID patients [4]. To the best of our knowledge, there is still a lack of information on cardiovascular outcomes in PACS. Thus, this narrative review scrutinized the available evidence, underscored the pathomechanisms responsible for acute COVID-19 that may also partake in long COVID, and formulated plausible hypotheses based on the existing evidence. Finally, we also aim to develop a comprehensive strategy for early detection and diagnosis of long COVID cardiovascular sequelae.

## 2. Post-Acute COVID-19 Syndrome (PACS)

Long COVID refers to the presence of numerous symptoms weeks or months after acquiring SARS-CoV-2 infection, regardless of viral state. It can be chronic or relapsing and remitting in nature, with the continuation of one or more acute COVID symptoms or the development of contemporary symptoms. Most persons with PACS tested negative for COVID-19, showing that the viral clearance in the body has been completely resolved. In other words, PACS is the period of time in which between microbiological and clinical recovery (with reference to both subjective, laboratory, and radiological findings) [5]. To avoid future ambiguity in describing this state across society, a uniform definition of long COVID has been established. According to the Centers for Disease Control and Prevention (CDC), PACS or long COVID is a condition in which new, continuous, or recurring symptoms arise four weeks or more after a COVID-19 infection. Moreover, PACS or long COVID may be separated into two stages based on the duration of symptoms: subacute/ongoing COVID, where symptoms last longer than 4 weeks but less than 12 weeks, and chronic COVID, where symptoms last longer than 12 weeks [6].

There are various difficulties in diagnosing long COVID. The period required for clinical recovery varies depending on the severity of the disease, and concomitant comorbidities make recognizing the cut-off point for diagnosis challenging. A considerable number of SARS-CoV-2 infected people are asymptomatic, and many people exhibit a wide range of clinical symptoms. If these people tend to develop several symptoms, later on, diagnosing long COVID will be quite complicated [7]. As a result, it is critical to better understand long COVID through a pathophysiologic concept in order to enhance understanding of a wide variety of clinical manifestations of long COVID for a diagnostic purpose.

## 3. Proposed Pathomechanisms of Long COVID in the Cardiovascular System

SARS-CoV-2 is already known to be responsible for the global COVID-19 pandemic on March 11, 2020 [8]. This entity resembles SARS-CoV-1 in many ways since both are positive-stranded RNA viruses with four structural proteins that anchor on the viral envelope [9]. Among these structural proteins, the spike (S) glycoprotein is the utmost important structure that is responsible for the host-cell entrance mechanism. The SARS-CoV-2 entrance pathway occurs when the S glycoprotein binds to the host cell’s angiotensin-converting enzyme-2 (ACE2) receptor, primarily in the type 2 pneumocytes, which results in viral membrane and host cell fusion [10]. The process is facilitated by the type II transmembrane serine protease (TMPRSS2) by activating the S protein. ACE2 receptors are ubiquitously expressed in various organs such as the lungs, intestines, kidneys, and importantly, the heart and endothelium [11]. Although both SARS-CoV-1 and 2 attach to the same receptors (ACE), enhanced infectivity has been observed in SARS-CoV-2. The reasons are twofold. To begin, SARS-CoV-2 has two-unit S glycoprotein, S1 and S2 [12]. Then, changes in the virus’s receptor binding region dramatically boosted SARS-CoV-2 affinity to ACE-2 by 10 to 20-fold over SARS-CoV-1 [13]. The heightened virulency of SARS-CoV-2 also translates to causing more harm as we highlighted later in the review.

**Fig. 1.**
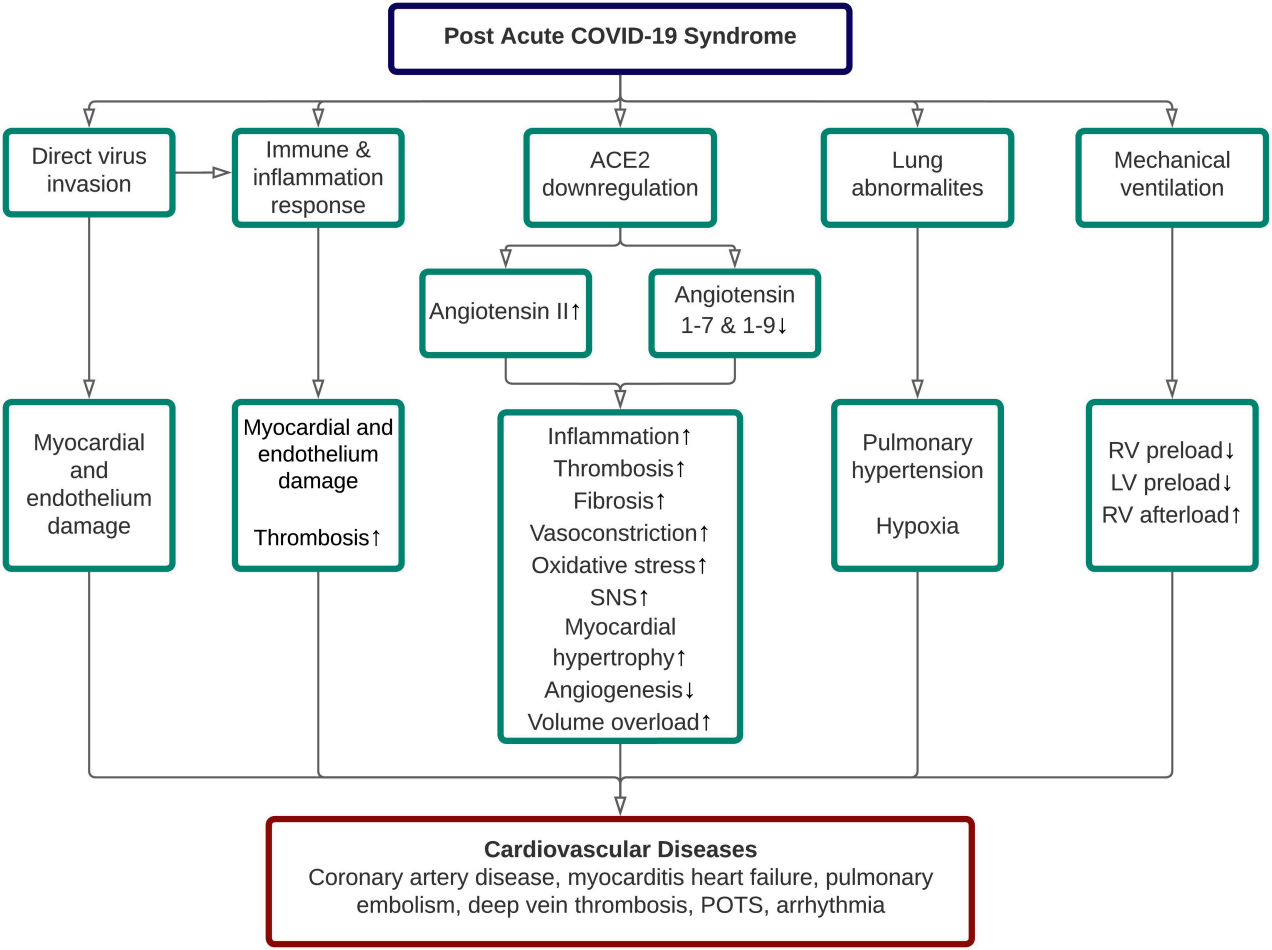
**Pathophysiology of cardiovascular diseases in post-acute COVID-19 syndrome**. ACE, angiotensin-converting enzyme; LV, left ventricle; POTS, postural orthostatic tachycardia syndrome; RV, right ventricle; SNS, sympathetic nervous system.

It has been generally known that the persistence of organ damage following an acute COVID-19 infection is related to PACS. Although several organs are affected and contribute to the persistence of symptoms in long COVID, we only highlight the cardiovascular (CV) sequelae of long COVID in this narrative review, primarily related to their possible underlying pathophysiology and modes for early detection. In general, five pathomechanisms contributed to the cardiovascular sequelae of long COVID, including direct SARS-CoV-2 invasion, aberrant immune and inflammatory response, ACE2 dysregulation, lung abnormalities, and adverse effect related to COVID-19 treatment itself [3]. The proposed pathophysiology of cardiovascular disease (CVD) in long COVID was depicted in Fig. 1.

### 3.1 Direct SARS-CoV-2 Invasion 

Direct invasion by SARS-CoV-2 into the heart and vessel-associated endothelial cells is possible due to the ACE-2 expression in these cells [12]. Several autopsy studies have identified the presence of SARS-CoV-2 in the heart and blood arteries, supporting this. According to a comprehensive assessment of 12 relevant studies that evaluated 105 post-mortem hearts, SARS-CoV-2 was found in nearly half of them (n = 50) [14]. Myocarditis (characterized as lymphocytic infiltration and necrosis of myocytes) is believed to be induced by the invasion of cardiomyocytes by SARS-CoV-2, thereby triggering innate and adaptive immune responses, resulting in cardiac inflammation through macrophage cytokines production and cell-mediated cytotoxicity [15]. This, in turn, will decrease heart function and, in the case of a chronic inflammatory state, may potentially result in fibrosis [16].

Varga *et al*. [17] discovered endothelial cell involvement in COVID-19 post-mortem cases. They discovered direct viral infection and subsequent inflammation of the endothelium. This inflammatory process induces immune cell recruitment, which causes endothelial dysfunction and vasoconstriction. This is followed by inadequate perfusion to organs and edema [17]. Eventually, endothelial injury also enhances the blood coagulation process by activating the tissue factors [18].

In a prospective cohort study, three patients who recovered 2–3 months after COVID-19 infection with severe myocarditis exhibited active lymphocytic inflammation and no evidence of any viral genome based on the endomyocardial biopsy [19]. Consistently, a cohort study by Zhan *et al*. [20] showed that in post-COVID-19 patients who remained positive by swab testing after various time points, viral replication and cytopathy effects, as evaluated by quantitative reverse transcription polymerase chain reaction (RT-qPCR) and cytopathy measurement, respectively, revealed no viral presence. Therefore, viral remnants were responsible for the positive swab result. As a result of these findings, we can fairly conclude that long-term negative effects in cardiovascular sequelae of long COVID patients are associated with the persistent viral reservoirs in the heart following the acute infection [20].

### 3.2 Aberrant Immune and Inflammatory Response

#### 3.2.1 Innate Immune Response

Once within the human body, any pathogen, including SARS-CoV-2, will elicit innate and adaptive immune responses. Activation of toll-like receptors 7 and 8 (TLR7 & TLR8), as well as NOD-like receptors (NLRs) on the surface of infected lung epithelial cells and alveolar macrophages, increases the production of type I and type III antiviral interferons (IFNs) and several distinct chemokines in the early phase of infection. These IFNs boost the expression of major histocompatibility complex (MHC) class I in additional infected cells, allowing CD8+ cytotoxic T cells and natural killer cells to block virus replication and restrict viral spread. Concurrently, other chemokines attract additional antigen-presenting cells (APCs) to the site of damage, such as dendritic cells, macrophages, and neutrophils, which then create more chemokines to recruit more CD4+ and CD8+ T cells. The virus will be presented to these lymphocytes by the APCs via class II MHC, and the APCs will also release pro-inflammatory cytokines such as interleukin-6 (IL-6) and tumor necrosis factor (TNF) [21, 22].

Recent research suggests that the innate immune response to SARS-CoV-2 differs from that of other viruses, such as its predecessor, SARS-CoV-1. An *in vitro* investigation conducted by Chu *et al*. [23] revealed that, whereas SARS-CoV-2 has a larger replication potential than SARS-CoV-1, it induces less IFN-I and IFN-III expression. However, it tends to dramatically stimulate several cytokines related to the inflammatory process, including interleukin-1β (IL-1β), interleukin-6 (IL-6), TNF, and interleukin-1 receptor antagonist (IL-1RA) based on an experimental study conducted by Blanco-Melo *et al*. [24].

#### 3.2.2 Adaptive Immune Response

APCs and infected host cells initiated the adaptive immune response by presenting the antigen to naive CD4+ helper T cells and CD8+ cytotoxic T cells via MHC class I and II, respectively. This entire process eventually resulted in cytotoxic factors lysis of the infected cells; activation of B cells, which produce specific antibodies to kill SARS-CoV-2; and secretion of numerous pro-inflammatory cytokines such as IFN-, IL-4, IL-5, and IL-13, which activate macrophages and create a vicious cycle resulting in the pathological inflammatory process [21, 22].

Several experimental investigations revealed that SARS-CoV-2 caused different adaptive immune responses as compared to other viral infections, such as the capacity to diminish lymphocyte numbers, resulting in a defective adaptive immune response and decreased viral clearance. A retrospective cohort research from Wuhan found that the major subsets of T lymphocytes, such as CD4+ and CD8+ T cells, are lowered in COVID-19 infection and are much lower in severe COVID-19 cases, as predicted [25]. Lymphopenia also significantly increased COVID-19 severity and mortality rate based on the meta-analysis conducted by Huang *et al*. [26]. Reduced lymphocyte generation with concomitant enhanced lymphocyte elimination is the primary pathomechanism causing lymphocyte decrease in COVID-19 infection SARS-CoV-2 can directly activate apoptosis in lymphocytes via the P-53 signaling pathway, resulting in enhanced lymphocyte elimination [27]. SARS-CoV-2 infects CD169+ macrophages in the spleen and lymph nodes (LNs), according to another investigation. As a result, splenic nodule atrophy and lymph follicle depletion occur, resulting in lymphoid tissue injury and a declension in lymphocyte production [28]. Commensurately, alterations in innate and adaptive immune responses are associated with the progression of viral infection, which can lead to uncontrolled inflammatory response, as indicated by increased production of pro-inflammatory cytokines, such as IL-6 [29, 30]. Consistently, pro-inflammatory cytokine such as IL-6 was elevated in critical-ill COVID-19 patients [31]. Ultimately, the uncontrolled inflammatory response can progress to a cytokine storm, which can cause myocardial damage and endothelial cell apoptosis [15, 32].

A significant inflammatory response to COVID-19 infection can potentially be harmful to the coagulation process. Animal research examining the relationship between CD8+ T cells and thrombosis in 11 HIV-uninfected subjects discovered that TNF-derived CD8+ T cells can increase tissue factor (TF) expression in vascular endothelium [33]. Furthermore, monocytes can express tissue factors through interactions with platelets via CD40L/CD40 binding. Antithrombin III (AT-III) and the protein C system generally control the pro-coagulant process. Nonetheless, neutrophils may use the elastase enzyme to break down AT-III and protein C. Proinflammatory cytokines such as IL-1 and TNF may inhibit thrombomodulin (TM), lowering protein C activation [34]. Taken together, these processes skewed the hemostatic balance to a thrombotic state, manifested in widespread microvascular thrombosis.

Persistent inflammation, as surrogated by the inflammation biomarkers in the long COVID patients such as C-reactive protein, procalcitonin, and IL-6 are seen in 8%, 4%, and 3% of long COVID, respectively [4]. Likewise, the local inflammation process in myocardial tissues caused, by direct SARS-CoV-2 infection could persist up to 2–3 months after the onset of infection in 60 out of 100 patients (60%). This persistent myocardial inflammation was detected using cardiovascular magnetic resonance (CMR) and confirmed in certain individuals by endomyocardial biopsies. To summarize, chronic inflammation is a possible underlying mechanism that led to cardiovascular problems in long COVID patients [19].

### 3.3 ACE2 Dysregulation

There are two primary converting enzymes in the renin-angiotensin-aldosterone system (RAAS), angiotensin-converting enzyme (ACE) and ACE2. Both enzymes were important in the acute COVID-19 pathomechanism. ACE2 degrades angiotensin II to angiotensin 1–7, as opposed to ACE, which converts angiotensin I to angiotensin II. Angiotensin I is also degraded by ACE2 into angiotensin 1–9 [35].

Angiotensin II will bind to angiotensin II receptor 1 (AT1R) and causes inflammation, fibrosis, increase oxidative stress, vasoconstriction, thrombosis, and reabsorption of sodium and water. Vascular leakage is the first phase to promote inflammation event. Angiotensin II via AT1R stimulates the production of prostaglandins (PGs) and vascular endothelial growth factor (VEGF) which is responsible for the increase in vascular permeability [36, 37]. Angiotensin II also promotes leukocyte recruitment and the production of proinflammatory cytokines such as interleukin-6 (IL-6), interleukin-8 (IL-8), and tumor necrosis factor-α (TNF-α) [37]. Angiotensin II can activate nicotinamide adenine dinucleotide phosphate (NADPH) oxidase in endothelial cells, resulting in an increase in reactive oxidative stress generation. Increased reactive oxidative stress buildup in the vasculature can alter the signaling cascade in cells, resulting in mitochondrial malfunction and, eventually, endothelial dysfunction [38]. Furthermore, a high level of reactive oxidative stress increases atherosclerosis and induces an inflammatory response [39]. Experimental animal studies showed that angiotensin II independently caused endothelial dysfunction by reducing the bioavailability of nitric oxide, resulting in vasoconstriction [40]. Angiotensin II can also upregulate collagen synthesis in cardiac fibroblasts, which induced the fibrotic process in the cardiac wall [41]. Several investigations have shown that angiotensin II can boost sympathetic nervous system activity by stimulating the brain, adrenal medulla, sympathetic ganglia, and sympathetic nerve terminal [42, 43, 44]. Angiotensin II also suppresses vagal activity by resetting baroreceptor reflex regulation, resulting in increased adrenergic activation [45]. Moreover, angiotensin II promotes thrombosis via various mechanisms, including increased tissue factor production via activation of nuclear factor kappa-B (NF-kB) and direct stimulations from AT1R binding, as well as coagulation activation via upregulation of prothrombin in the plasma [46, 47]. Furthermore, angiotensin II inhibits the fibrinolysis process via activation of the plasminogen activator inhibitor 1 (PAI-1) which reduced plasma plasmin levels [48]. Lastly, angiotensin II via AT1R promotes aldosterone release from kidneys, thus increasing the sodium and water reabsorption from proximal tubules [49, 50].

Angiotensin 1–7 and 1–9, on the other hand, induce various beneficial cardiovascular actions related to cardiac and vascular remodeling. In-vitro investigations on mice revealed that angiotensin 1–7 can suppress collagen and fibronectin deposition and proliferation, hence preventing cardiac fibrosis [51, 52]. Angiotensin 1–7 has also been shown to reduce collagen proliferation by inhibiting cardiac fibroblast collagen production via extracellular-signal-regulated kinase (ERK) phosphorylation [53]. Furthermore, angiotensin 1–7 has a potential role to attenuate atrial tachycardia events by (1) decreasing the potential action duration via reduced expression of L-type calcium channel and outward potassium channel [54]; (2) preventing fibrotic process in the atrial wall, which predisposed to atrial tachycardia [54]; and (3) decreasing the norepinephrine release from hypothalamus, which resulted in the attenuation of sympathetic stimulation [55]. Angiotensin 1–7 can also prevent myocardial hypertrophy, and left ventricular thinning, and reduce myocardial infarct area in the post-MI setting [56]. Few studies also support that angiotensin 1–7 can prevent myocardial hypertrophy by inhibiting the growth of myocardial cells [57, 58, 59]. Additionally, angiotensin 1–7 may also have an anti-inflammatory effect by increasing the level of anti-inflammatory cytokines, including interleukin 10 (IL-10), and reducing the expression of pro-inflammatory cytokines such as IL-6 and tumor necrosis factor-α (TNF-α) [56]. Based on another experimental study, angiotensin 1–7 tends to inhibit the production of cardiac reactive oxygen species (ROS) and improve endothelial function by increasing nitric oxide production, which resulted in vasodilatation [60, 61]. An increased level of nitric oxide is achieved by activating the endothelial nitric oxide synthase (eNOS) through direct stimulation of angiotensin 1–7 on the bradykinin kinase 2 receptor (BK2R) and angiotensin 1–7 on the angiotensin 2 receptor (AT2R) pathway [62]. Moreover, an in-vitro study revealed that angiogenesis can be promoted by angiotensin 1–7 via increased formation of vascular endothelial growth factor D (VEGF-D) and matrix metalloproteinase-9 (MMP-9) [63]. Angiotensin 1–7 also plays a role in reducing atherosclerotic lesion burden, acts as a plaque stabilization, and has anti-thrombotic properties [64, 65, 66]. In a similar fashion to angiotensin 1–7, angiotensin 1–9 can prevent myocardial hypertrophy, attenuate the myocardial cell fibrotic process, and promote vasodilatation via the AT2R signaling pathway [67, 68].

SARS-CoV-2 interaction with the ACE2 receptor results in ACE2 downregulation. As a result, the amount of angiotensin II rises while the levels of angiotensin 1–7 and angiotensin 1–9 fall. However, to the best of our knowledge, there is no literature on ACE2 dysregulation and its impact on long COVID patients. To summarize, ACE downregulation causes a slew of negative downstream consequences due to decreased protective effects of angiotensin 1–7 and 1–9 and unopposed angiotensin II functions, resulting in a deterioration of the patient’s state through a variety of cardiovascular problems [24].

### 3.4 Lung Abnormalities 

Acute SARS-CoV-2 infection can result in severe lung damage, respiratory dysfunction, hypoxia, and hypoxemia [69]. Respiratory dysfunction persists, recurs, or has recently happened in numerous individuals with protracted COVID-19. A meta-analysis also showed that 34% of long COVID patients have abnormal chest X-rays/CT scans in the lungs [4]. Hypoxia can result in type II myocardial infarction due to demand ischemia [70]. Moreover, hypoxia can promote anaerobic metabolism, which induces intracellular acidosis, resulting in lactic acid accumulation [71]. Mediated by the hypoxia-inducible factor-1 (HIF-1) site, this acidotic state activates the protein of death-promoting BCL2 adenovirus E1B 19 kDa protein-interacting protein 3 (BNIP3) gene resulting in myocardial cell death [72].

SARS-CoV-2 infection can induce ACE2 downregulation in the lungs, increasing the angiotensin II and diminishing the protective effects of angiotensin 1–7 and angiotensin 1–9. Consequently, this cascade causes pro-inflammatory cytokines upregulation and increases vascular permeability, promoting endothelial dysfunction, endothelial cell proliferation, and vasoconstriction in the lungs [73]. These processes also affect the pulmonary arteries and lead to pulmonary vascular remodeling and hypertension [73, 74]. Finally, pulmonary hypertension-induced vascular wall stiffness can increase the right ventricular (RV) afterload and precipitates RV wall stress [75].

### 3.5 Acute COVID-19 Treatment

#### Invasive Mechanical Ventilation

A substantial number of severe and critical COVID-19 patients need mechanical ventilation to support ventilation and gas exchange in the alveoli [76]. Nonetheless, there are cardiac complications associated with mechanical ventilation use, primarily to the right ventricle (RV) and the left ventricle (LV). Generally, mechanical ventilation could decrease the RV preload and concurrently increase the RV afterload [77]. The mechanisms are described as follows. During regular inspiration, there is a decrease in intrathoracic pressure (ITP). This pressure is transmitted to the right atrium through the pericardium and decreases the right atrial pressure (RAP), thus decreasing venous return [78]. In contrast, when a patient is on mechanical ventilation with high positive end-expiratory pressure (PEEP), the ITP increases and decreases the venous return [77]. In addition, PEEP can also increase the RV afterload and decrease the LV preload by increasing pulmonary vascular resistance [79].

An experimental study by Ross *et al*. [80] showed that the stroke volume and cardiac index were significantly lower at a PEEP of 15 ${\mathrm{cmH}}_{2}$O compared to a PEEP of 5 ${\mathrm{cmH}}_{2}$O. Another cohort study by Hill *et al*. [81] showed the long-term outcomes after prolonged invasive mechanical ventilation in critically ill patients. Compared to patients who underwent invasive mechanical ventilation shorter than 21 days, those who needed invasive mechanical ventilation (IMV) for more than 21 days are at increased risk of readmission to the intensive care unit (ICU) (adjusted OR: 1.20; 95% CI: 1.14–1.26) and rehospitalization (adjusted OR: 1.49; 95% CI: 1.39–1.60) [81].

In a retrospective cohort study, a large variation of PEEP levels and duration of IMV support in acute COVID-19 patients is seen in those who survived the disease [82]. The PEEP levels ranged from 5 ${\mathrm{cmH}}_{2}$O to 28 ${\mathrm{cmH}}_{2}$O, with an average PEEP level of 12 ${\mathrm{cmH}}_{2}$O. The duration of mechanical support ranged from 1 to 59 days, with an average of 14.6 days (±12 SD). Thus, considering the detrimental effect of IMV on cardiovascular physiology, a subset of patients is expected to experience cardiovascular complications. Consistently, a retrospective cohort study revealed that those who received IMV are at an increased risk to suffer long COVID compared to those who did not receive supplemental oxygen (OR: 2.42, 95% CI: 1.15–5.08) [83]. Thus, COVID-19 patients who needed IMV and experiencing PACS will almost certainly require further cardiovascular examination.

## 4. Possible Role of Cardiovascular Disease Related Comorbidities in the Genesis of Long-COVID 19 Syndrome

The significant morbidity, mortality, and poor outcomes associated with PACS connected to cardiovascular disease have piqued the attention of the medical community in characterizing CVD consequences in long COVID. As a result, findings from prospective observational studies will continue to impact our knowledge of the long-term implications outlined above. In this review, we included 17 prospective observational studies with a total of 8450 COVID-19 participants who were followed up on for about 9.3 months. Table 1 (Ref. [20, 83, 84, 85, 86, 87, 88, 89, 90, 91, 92, 93, 94, 95, 96, 97, 98]) shows the baseline characteristics of the included studies.

**Table 1.** **General characteristics of the included prospective observational studies**.

No.	Author (year)	Country	Study population	Age (years)	Male (%)	Cardiovascular comorbid(s) (%)	Cardiovascular-related symptoms (%)	Pathologic cardiovascular-related diagnosis/laboratory/imaging findings	Follow-up time (month(s))
1	Catalán *et al*. (2021) [84]	Spain	76 hospitalized COVID-19; 22.8% severe illness	65 ± 9.7	63.7	Hypertension: 53.9	Chest pain: 11.4	N/A	12
						Diabetes: 10.5	Dyspnea: 25		
						Dyslipidemia: 39.5	Fatigue: 51.3		
						Obesity: 61.8			
						Atrial fibrillation: 3.9			
						Smoking: 5.2			
2	Fernández‑de‑las‑Peñas *et al*. (2021) [85]	Spain	2100 hospitalized COVID-19; 6.6% severe illness	61 ± 16	53.1	Hypertension: 26.4	Chest pain: 6.5	N/A	11.2
						Diabetes: 12.1	Dyspnea: 23.4		
						Obesity: 45.1	Fatigue: 61.4		
						CVD (unspecified): 12			
3	Gamberini *et al*. (2021) [86]	Italy	470 hospitalized COVID-19; 100% severe illness	64 ± 7.8	72.5	Hypertension: 49.4	Dyspnea: 58.4	N/A	12
						Diabetes: 15.7	Fatigue: 74.6		
						CVD (unspecified): 7.3	Palpitations: 6.7		
4	Huang *et al*. (2021) [83]	China	1276 hospitalized COVID-19; 4% severe illness	59 ± 9.1	53	Hypertension: 36	Chest pain: 7	N/A	12
						Diabetes: 15	Dyspnea: 49		
						CAD: 8	Fatigue: 52		
						Smoking: 7	Palpitations: 10		
5	Liu *et al*. (2022) [87]	China	594 hospitalized COVID-19; 14% severe illness	63 ± 5	46.3	Hypertension: 37.4	Chest pain: 1	Laboratory:	12
						Diabetes: 17.3	Dyspnea: 2.7	- Increased cardiac troponin: 0.05%	
						CVD (unspecified): 6.2	Fatigue: 3.7	- Increased NT-pro BNP: 14.2%	
						Smoking: 5.9	Palpitations: 1.6	- Increased D-dimer: 2.7%	
6	Maestre-Muñiz *et al*. (2021) [88]	Spain	445 hospitalized COVID-19	71.5 ± 14.3	45.2	Hypertension: 67.4	Chest pain: 53.3	N/A	12
						Diabetes: 33.7	Dyspnea: 49.6		
						Obesity: 68.1	Fatigue: 65.9		
						CAD: 13.3	Palpitations: 60.9		
7	Maestrini *et al*. (2021) [89]	Italy	152 hospitalized COVID-19; 29% severe illness	69 ± 11.2	52.6	Hypertension: 33.9	Chest pain: 1.7	New-onset hypertension: 6.5%	12
						Diabetes: 15.8	Dyspnea: 10.8	Echocardiography:	
						Obesity: 27	Fatigue: 14.2	- LV dysfunction: 47.6%	
						CAD: 13.1	Palpitations: 4.2	- RV dysfunction: 14.3%	
						HF: 7.9		- PH: 3.2%	
8	Méndez *et al*. (2022) [90]	Spain	171 hospitalized COVID-19; 18.7% severe illness	58 ± 8.6	57.9	Hypertension: 32.2	Chest pain: 7.6	N/A	12
						Diabetes: 14.6	Dyspnea: 25.7		
						CVD (unspecified): 4.7	Fatigue: 48.5		
						Smoking: 5.8			
9	Myhre *et al*. (2021) [91]	Norway	58 hospitalized COVID-19; 19% severe illness	56 ± 11.3	58.6	Hypertension: 21.1	Chest pain: 4	Laboratory:	6
						Diabetes: 10.3	Dyspnea: 55	- Increased cardiac troponin: 10%	
						Obesity: 24.1	Fatigue: 64	- Increased NT-pro BNP: 12%	
						CVD (unspecified): 8.6		CMR:	
						Smoking: 1.8		- LGE: 17%	
10	Puntmann *et al*. (2020) [92]	Germany	100 hospitalized COVID-19; 2% severe illness	49 ± 14	53	Hypertension: 22	Chest pain: 17	Laboratory:	2.3
						Diabetes: 18	Dyspnea: 36	- Increased cardiac troponin: 5%	
						Dyslipidemia: 22	Palpitations: 20	CMR:	
						CAD: 13		- LGE: 10%	
						Smoking: 22			
11	Raman *et al*. (2021) [93]	United Kingdom	58 hospitalized COVID-19; 36.2% severe illness	55.4 ± 13.2	58.6	Hypertension: 37.9	Chest pain: 27.6	CMR:	3
						Diabetes: 13.8	Dyspnea: 87.9	- Myocarditis: 11.5%	
						Obesity: 81			
						CAD: 3.4			
						Smoking: 34.5			
12	Seeßle *et al*. (2022) [94]	Germany	96 hospitalized COVID-19; 4% severe illness	57 ± 6.8	44.8	Hypertension: 35.1	Dyspnea: 27.1	N/A	12
						Diabetes: 7.3	Fatigue: 41.7		
						Obesity: 34			
						CVD (unspecified): 4.2			
13	Sonnweber *et al*. (2021) [95]	Austria	109 hospitalized COVID-19; 27% severe illness	57 ± 14	57	Hypertension: 30	Dyspnea: 36	Laboratory:	3.3
						Diabetes: 17		- Increased NT-pro BNP: 11%	
						Dyslipidemia: 19		Echocardiography:	
						CVD (unspecified): 40		- LV dysfunction: 3%	
						Smoking: 3		- Myocarditis: 6%	
								- PH: 10%	
14	Zhan *et al*. (2021) [20]	China	121 hospitalized COVID-19; 16% severe illness	50 ± 10.2	41.3	Hypertension: 25.6	Dyspnea: 18.2	New-onset hypertension: 31.6%	12
						Diabetes: 6.6	Fatigue: 11.6	Laboratory:	
						CVD (unspecified): 2.5		- Increased NT-pro BNP: 5.3%	
								ECG:	
								- Arrhythmia (unspecified): 15.8%	
								Echocardiography:	
								- LV dysfunction: 31.6%	
								- RV dysfunction: 16.7%	
								CMR:	
								- LGE: 33%	
15	Zhang *et al*. (2021) [96]	China	2433 hospitalized COVID-19; 28% severe illness	60 ± 9.7	49.5	Hypertension: 29.3	Chest pain: 13	N/A	12
						Diabetes: 13.9	Dyspnea: 2.7		
						CVD (unspecified): 9.2	Fatigue: 27.7		
						Smoking: 6.4	Palpitations: 4.2		
16	Zhao *et al*. (2021) [97]	China	94 hospitalized COVID-19; 46% severe illness	48.1	57.5	Hypertension: 17	Chest pain: 13.8	N/A	12
						Diabetes: 9.6	Fatigue: 39.4		
						CVD (unspecified): 4.3	Palpitations: 11.7		
17	Zhou *et al*. (2021) [98]	China	97 hospitalized COVID-19; 0% severe illness	46.5 ± 18.6	53.6	Hypertension: 24.7	Dyspnea: 8.2	Laboratory:	1
						Diabetes: 11.3		- Increased cardiac troponin: 6.2%	
						CAD: 6.2		- Increased NT-pro BNP: 0.9%	
								ECG:	
								- Atrial fibrillation 1%	
								Echocardiography:	
								- LV dysfunction: 1%	

CAD, coronary artery disease; CMR, cardiac magnetic resonance; CVD, cardiovascular disease; ECG, electrocardiography; HF, heart failure; LGE, late gadolinium enhancement; LV, left ventricle; MV, mechanical ventilation; N/A, not available; NT-pro BNP, N-terminal pro-B type natriuretic peptide; RV, right ventricle.

Research is emerging on predictors for long COVID. We postulate that the presence of PACS in some but not all patients is due to a combination of characteristics that contribute to chronic inflammation in long COVID, such as the severity of acute COVID-19, obesity, hypertension, diabetes mellitus, and age.

Because of the hyperinflammatory condition and substantial tissue damage, severe acute COVID-19 is a risk factor for long COVID, as seen in Table 1, with 23.3 percent of individuals experiencing long COVID coming from the severe disease group. A retrospective cohort research also found that individuals who required non-invasive ventilation (NIV) and IMV were more likely to have long COVID than those who did not (OR: 2.42, 95% CI: 1.15–5.08) [83]. Furthermore, a retrospective study conducted by Sonaglioni *et al*. [99] showed that Charlson Comorbidity Index (CCI) ≥7, neutrophil-to-lymphocyte (NLR) ratio ≥9, and undertreatment with angiotensin-converting enzyme inhibitors (ACEIs) or angiotensin receptor blockers (ARBs), were independently associated with a higher risk of in-hospital mortality in hospitalized COVID-19 patients. It denotes that COVID-19 patients with a higher number of comorbidities, prominent inflammatory state, and RAAS activation were more likely to present with the severe course which subsequently resulted in a higher risk of long COVID-19 [99].

**Fig. 2.**
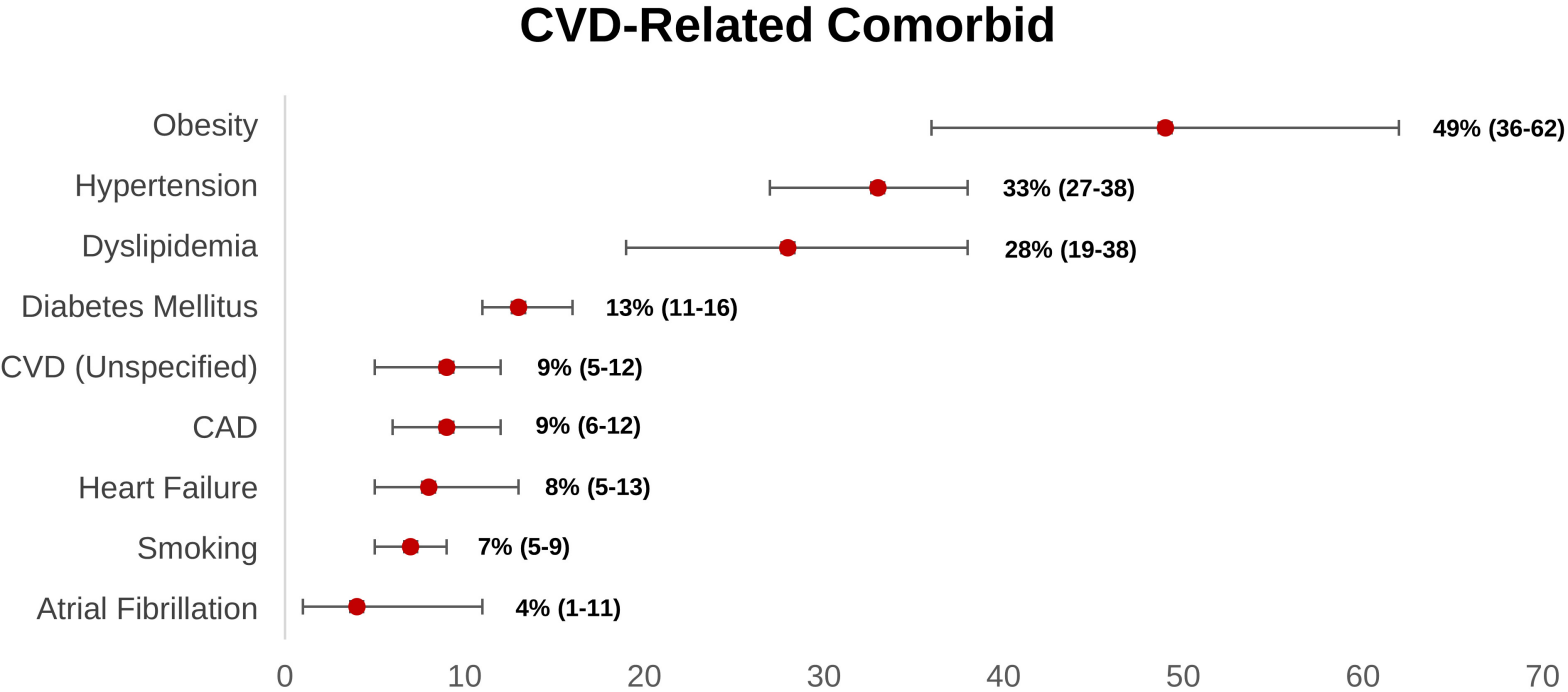
**Weighted prevalence of CVD-related comorbid (95% CI) reported in post-acute COVID-19 syndrome patients**. CAD, coronary artery disease; CI, confidence interval; CVD, cardiovascular disease.

Obesity is the most prevalent cardiovascular-related comorbidity reported within the long COVID group, according to our research (Fig. 2). Obese people have greater levels of pro-inflammatory cytokines (tumor necrosis factor- (TNF-), IL-6, and so on) due to adipocyte overexpression [31, 100].

Obesity also influenced the innate immune system, as seen by the increased inflammatory response [101]. Consistently, Tenforde *et al*. [102] also demonstrated that acute COVID-19 patients with obesity are more likely to have persistent symptoms 14–21 days following the COVID-19 diagnosis (*p*-value: 0.002; adjusted OR: 2.31, 95% CI: 1.21–4.42). SARS-CoV-2 might enter the cell via ACE2-Spike binding and Spike priming by host cell TMPRSS2. TMPRSS2 involves proteolytical protein breakdown and folding to a post-fusion conformation, as well as host cell-virus membrane fusion and cytosolic viral RNA release. Interestingly, there is an increase in glycated ACE2 and TMPRSS2 expression in obese individuals. As a result, vulnerability to SARS-CoV-2 infection and its link to poor prognosis appears to be increased in this group [103, 104]. Moreover, a systematic review conducted by Boroumand *et al*. [105] showed that higher BMI was associated with a lower antibody response after COVID-19 vaccination. Given the significant link between obesity and long COVID, weight-loss interventions such as calorie restriction, diet, exercise, and stress reduction may be effective in reducing an overexpression of ACE2 in cardiomyocytes, increasing immune response after administration of COVID-19 vaccines, and lowering the risk of CVD-related illness in long COVID [106].

Weighted prevalence data from 17 prospective observational studies showed that hypertension was the second most common comorbid in long COVID patients. In the acute setting, a meta-analysis by Du *et al*. [107] revealed that hypertension independently and significantly increased the risk of severe course and in-hospital mortality in COVID-19 patients. In a molecular perspective, because of hypertension, there is a systemic inflammatory response, characterized by the activation of complement, myeloid cells, inflammasome, and changes to the vascular cells. Consequently, these conditions lead to renal and vascular dysfunction, which worsens blood pressure elevation and leads to end organ damage. Hence, theoretically, hypertension could enhance the chronic inflammatory response in acute COVID-19 patients, resulting in long COVID [108]. Consistently, this hypothesis was supported by a case-control study by Fernández-de-las-Peñas *et al*. [109] that revealed preexisting hypertension was linked with a more significant number of long COVID symptoms compared to those without hypertension. Regarding the use of anti-hypertensive drugs, a meta-analysis conducted by Ren *et al*. [110] demonstrated that prior utilization of antihypertensive drugs (e.g., ACEIs/ARBs, calcium channel blockers, beta-blockers, or diuretics) was not substantially correlated with the risk and severity of COVID-19. Additionally, in sub-group analysis, the risk of severe COVID-19 and mortality were significantly decreased in hypertensive patients who taking ACEIs/ARBs [110]. However, a prospective longitudinal study by Sardu *et al*. [111] revealed that there were no significant differences in detrimental outcomes (ICU admission, MIV, and mortality) in COVID-19 patients with hypertension who receive ACEIs, ARBs, and calcium channel blockers (CCB). Furthermore, a longitudinal study by Soegiarto *et al*. [112] showed that hypertension patients presented with lower antibody response and recurrent COVID-19 infection after COVID-19 vaccination. Fascinatingly, patients with non-O blood group showed greater prothrombotic index values and a higher incidence of cardiac injury and mortality [113]. Hence, these occurrences may explain why individuals with hypertension and COVID-19 have a poor prognosis. Regardless of the class of anti-hypertensive drugs, optimal blood pressure control was recommended as it can reduce the probability of hypertensive patients suffering recurrent COVID-19, severe COVID-19, and long COVID.

Diabetes mellitus (DM) also contributed to the development of long COVID, which accounts for 13% long COVID patients had DM. Two meta-analyses found that patients with a history of DM or acute hyperglycemia at admission significantly increased the risk of severe COVID-19 and mortality [114, 115]. In diabetic patients, there are dysregulation of glucose hemostasis, reduced immune modulation, hyperinflammatory response, and RAAS activation. Hence, when COVID-19 infection occurs, it can lead to endothelial damage, increased oxidative stress and pro-inflammatory cytokines, and glucotoxicity, resulting in multi-organ dysfunction, increased of thromboembolic risk, lung fibrosis, and acute respiratory distress syndrome, which consequently ended in severe COVID-19 and increases the risk of long COVID [116, 117, 118]. Herman-Edelstein *et al*. [103] study performed the biopsy of the right atrial appendage in 76 patients (57 diabetic patients and 22 non-diabetic patients). This study revealed that diabetic patients had an up-regulation of ACE2 receptors in heart tissue compared to non-diabetic patients, and higher HbA1c levels were correlated with overexpression of ACE2 receptors in cardiomyocytes [103]. It underscores that diabetic patient had a higher possibility of CVD induced by COVID-19 infection distinctive to nondiabetic patients. Furthermore, like hypertension, diabetes can also alter the immunogenicity of COVID-19 vaccines. A prospective observational study conducted by Marfella *et al*. [116, 117] suggested that hyperglycemia at the time of vaccination worsens the immunological response and achieving appropriate glycemic control during the post-vaccination period improves the immunological response. Therefore, adequate glycemic control in diabetic patients is warranted as it increased the antibody response after COVID-19 vaccination, decreased the overexpression of ACE2 in cardiomyocytes, and reduced the risk of severe COVID-19 as well as long COVID.

Another hypothesis for persistent inflammation in long COVID patients is that they are older, which is substantiated by the fact that the majority of long COVID patients in our review were elderly (Table 1) (mean age: 60.2 years old). According to an animal investigation, aged mice had refractory interferon activity in alveolar macrophages and elevated pro-inflammatory cytokine output [119]. They also have reduced B cell response, lower plasma cell synthesis in the bone marrow, and lower naive T cell output due to age-related thymus atrophy [120]. In addition, like the obese and diabetic population, older age patient also presented with lower antibody response after COVID-19 infection [105]. Taken together, these mechanisms are likely represented in older COVID-19 patients, preventing full viral clearance, and resulting in viral progression and enhanced inflammatory response. A cohort study also revealed that elderly patients with acute COVID-19 are at a higher risk of persistent symptoms up to 14–21 days after the COVID-19 diagnosis (*p*-value: 0.010; adjusted OR: 2.29, 95% CI: 1.14–4.58) [102].

## 5. Symptoms and Pathologic Findings of Cardiovascular Disease in Post-Acute COVID-19 Syndrome

### 5.1 Symptoms

**Fig. 3.**
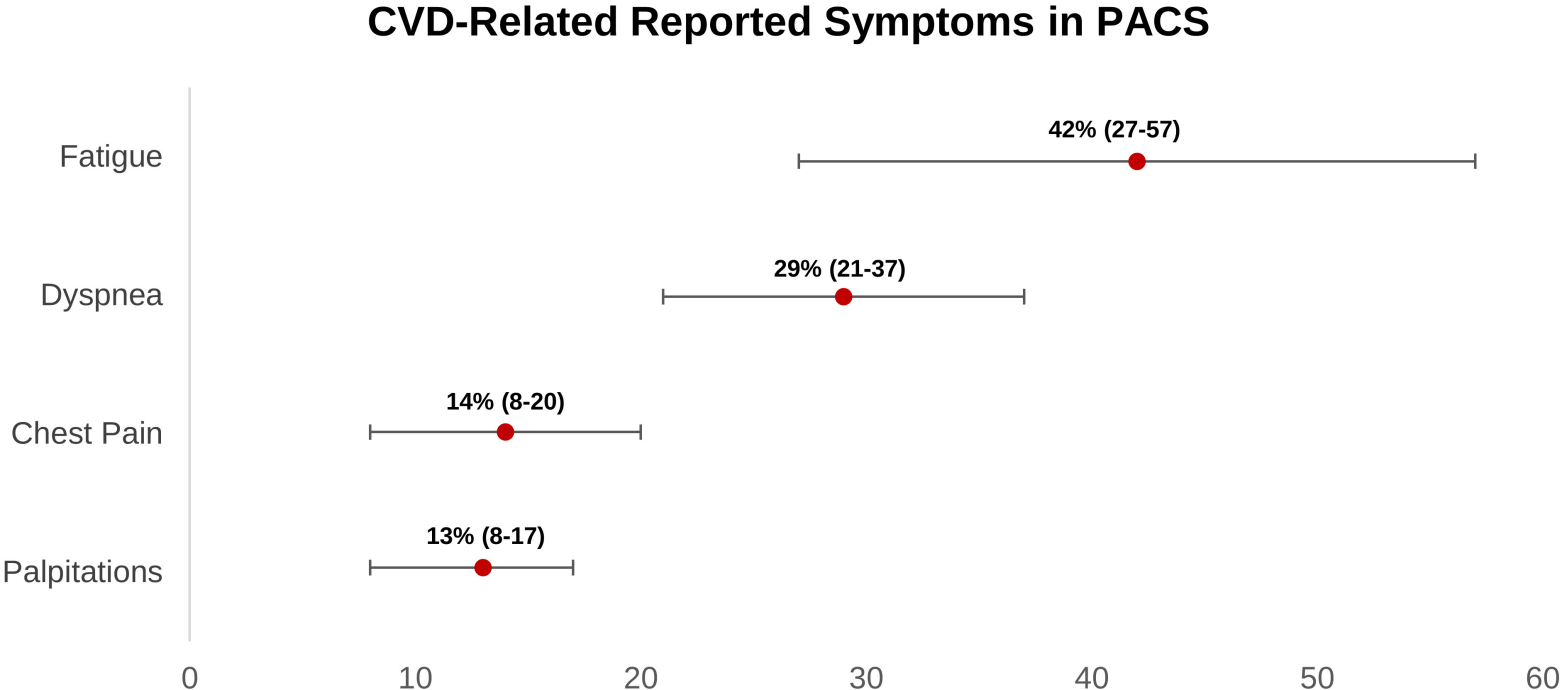
**Weighted prevalence of CVD-related symptoms (95% CI) reported in post-acute COVID-19 syndrome patients**. CI, confidence interval; CVD, cardiovascular disease; PACS, post-acute COVID-19 syndrome.

According to our findings, four major symptoms would arise in long COVID individuals who had a cardiovascular sequela. Fatigue is the most prevalent symptom, followed by dyspnea, chest pain, and palpitations (Fig. 3). According to twelve cohort studies, the prevalence of chest pain in long COVID ranges from 8% to 20%. There are no cohort studies that have thoroughly examined the features of chest discomfort in COVID-19 patients who have been on the drug for a long time. Thus, long COVID chest discomfort can be caused by a variety of conditions, including cardiovascular diseases such as pulmonary embolism, coronary artery disease, and myocarditis. In these CVDs, chest discomfort may be caused by nerve-ending activation (C7-T4) caused by lactate and adenosine buildup in ischemic myocardial cells [84, 85, 87, 88, 89, 90, 91, 92, 93, 96, 97, 121].

Regarding palpitations, based on our findings, it occurs in 13% (8%–17%) of long COVID patients. Palpitations can arise as a result of increased sympathetic tone in heart failure patients or as a result of another tachyarrhythmia. Patients with long COVID mostly experience fatigue ranging from 27 percent to 57 percent. Fatigue can arise as a result of skeletal muscle oxygen perfusion loss, which happens in nearly all CVD patients, including heart failure, coronary artery disease, pulmonary embolism, myocarditis, arrhythmia, and postural orthostatic tachycardia syndrome (POTS) [85].

Related to dyspnea, it occurs in 21%–37% of patients with long COVID. Lung edema caused by heart failure or pulmonary arteries occlusion caused by pulmonary embolism, present with dyspnea. Other non-specific symptoms that could occur in long COVID are limb edema, cough, nausea, vomiting, depression, and sweating. Based on a meta-analysis, increased D-dimer and NT-pro BNP levels were also found in 20% and 11%, respectively, in long COVID patients. These two biomarkers are commonly elevated in venous thromboembolism and heart failure patients, respectively [4].

### 5.2 Pathologic Findings

#### 5.2.1 Coronary Artery Disease (CAD)

The incidence of coronary artery disease in long COVID is unclear, in contrast to the acute situation. Two case series studies revealed that there were 20.4 to 38% of COVID-19 patients with ST-elevation myocardial infarction (STEMI) who had coronary artery obstruction confirmed by coronary angiography and presented with no chest pain at admission [122, 123]. Moreover, Bangalore *et al*. [123] study showed that there were 46% of COVID-19 patients develop STEMI during hospitalization. Thus, it underscores that COVID-19 can lead to systemic inflammatory response syndrome and eventually increases the risk of plaque rupture, thrombus formation, and endothelial dysfunction, resulting in acute coronary syndrome [124].

Nonetheless, up to 20 percent of long COVID patients experience chest discomfort. Several cardiovascular investigations, including electrocardiography, laboratory testing (such as troponin and creatine kinase-myocardial band (CK-MB)), a treadmill test, cardiac CT, and angiography, can aid in the diagnosis of CAD [125, 126, 127]. In theory, long COVID has two pathogenic mechanisms: direct invasion and ACE2 downregulation, both of which can lead to coronary artery disease. SARS-CoV-2 invasion into the vasculature induced direct endothelial damage, resulting in endothelial dysfunction, inflammation, and vasoconstriction. Furthermore, angiotensin II can activate platelets and disrupt the anticoagulant process [46, 47, 48]. Simultaneously, lower levels of angiotensin 1–7 and angiotensin 1–9 lowered their anti-thrombotic, plaque stabilization, and vasodilatory activities [64, 65, 66, 67]. These mechanisms, when combined, might aggravate the underlying atherosclerotic lesions in the coronary artery. In addition, macrophage activation by an immunological response can release collagenases, which can destroy the interstitial collagen of a fibrous cap. Finally, the vasoconstriction that raises blood velocity through the weaker fibrous cap might produce a plaque rupture and lead to acute coronary syndrome [128]. Alternatively, demand ischemia caused by hypoxia in long COVID patients is also plausible pathophysiology that leads to type II myocardial infarction [70].

#### 5.2.2 Venous Thromboembolism (VTE)

To the best of our knowledge, no studies have investigated the performance of ultrasonography or computed tomography of the pulmonary artery (CTPA) as a diagnostic tool for venous thromboembolism in patients with long COVID. According to a meta-analysis of observational studies, increased D-dimer levels were seen in 134 of 359 (20%) long COVID patients. While it has a high sensitivity for excluding deep venous thrombosis (DVT) and pulmonary embolism (PE) (84 and 99.5 percent, respectively) [129], the D-dimer specificity to diagnose DVT and PE are much lower (50% and 41%, respectively) [130]. Thus, increased D-dimer level raises the suspicion of venous thromboembolism. The diagnosis of PE is established by a laboratory test, chest X-ray, echocardiography, and CTPA [131]. Whereas the diagnosis of DVT is established through laboratory tests and doppler ultrasonography [132]. Venous thromboembolism could occur in long COVID because of a thrombogenic, hypercoagulable state, and endothelial dysfunction, due to the direct invasion of endothelial cells by SARS-CoV-2 and ACE2 dysregulation.

#### 5.2.3 Heart Failure

Although the incidence of heart failure in long COVID patients is unclear, our data revealed that high NT-pro BNP levels were found in 6% of long COVID patients. Long COVID patients may experience heart failure-related symptoms such as dyspnea, palpitations, tiredness, and limb edema [4]. Electrocardiography, laboratory tests (such as NT-pro BNP), chest x-rays, and echocardiography are all useful diagnostic methods for determining heart failure [133]. Heart failure has complicated pathophysiology that includes problems in preload, contractility, and afterload. In critical acute COVID-19, invasive mechanical ventilation can limit venous return and increase intrathoracic pressure, leading to RV preload reduction and RV afterload elevation [77]. Elevated RV afterload can potentially result in pulmonary hypertension through pulmonary vascular remodeling [75]. Direct SARS-CoV-2 myocardial cell invasion leads to myocarditis, which impairs heart contractility. In addition, type II myocardial infarction induced by hypoxia and type I myocardial infarction caused by coronary artery occlusion also reduce myocardial contractility [15].

#### 5.2.4 Postural Orthostatic Tachycardia Syndrome (POTS)

The available data on POTS in long COVID is still sparse. According to four case reports regarding POTS, it develops in young adults with previously mild-moderate COVID-19. Generally, the symptoms, including palpitations, chest pain, dyspnea, and fatigue, are provoked by standing. Additionally, based on two case reports, adrenaline surge-related symptoms occur in patients such as dry mouth, diarrhea, and tremor. Tachycardia on standing without orthostatic hypotension is also seen in all case reports. The diagnosis of POTS was established through variable autonomic function tests, including head-up tilt table test (HUTT), quantitative sudomotor axon reflex testing (QSART), heart rate variability with standing, deep breathing, and Valsalva maneuver [134, 135, 136, 137].

Hypothetically, POTS is caused by autonomic dysfunction in long COVID patients [138]. Autonomic dysfunction in long COVID is provoked by the hyperadrenergic state and the resetting of baroreceptor control, which is stimulated by angiotensin II upregulation. This hypothesis is supported by a case reported by Umapathi *et al*. [134]. In the case report, increased urinary catecholamine was seen in a long COVID patient with POTS [134]. Conversely, another case by Miglis *et al*. [137] did not find elevated plasma norepinephrine levels in a long COVID patient with POTS. Thus, the exact pathophysiology of POTS in long COVID is still undetermined, and further research is needed.

#### 5.2.5 Hypertension

Our findings found that the prevalence of new-onset hypertension in long COVID is 19.1%. Hypertension could be explained due to ACE2 downregulation. Increased angiotensin II levels can also cause endothelial dysfunction via several pathways, resulting in reduced nitric oxide (NO) bioavailability and leading to vasoconstriction [38]. Additionally, angiotensin II can also cause reactive oxidative stress accumulation and inflammation in the vasculature, which accelerates atherosclerosis. Simultaneously, decreased angiotensin 1–7 levels exaggerate the pathological processes due to diminishing counter-regulatory effects. The culmination of vasoconstriction and atherosclerosis is new-onset hypertension [37, 39].

#### 5.2.6 Myocarditis 

Our study revealed that 7% of long COVID diagnosed with myocarditis. Of note, a cohort study by Puntmann *et al*. [19] showed that myocarditis could persist until 2–3 months after the onset of infection in 60 out of 100 patients. Alarmingly, this chronic inflammation process also caused pericardial effusion in 10 out of 100 long COVID patients. Furthermore, high-sensitivity troponin T values were increased (≥3 pg/mL) in 71 patients (71%) and significantly elevated (≥13.9 pg/mL) in 5 patients (5%) [19].

The gold standard for myocarditis diagnosis is the endomyocardial biopsy, but CMR can be a valuable alternative to evaluate abnormalities in the cardiac wall due to myocarditis because it is a non-invasive diagnostic tool [139, 140]. Additionally, cardiac troponin T or CK-MB will give information regarding the extent of myocyte damage and aid the myocarditis diagnosis [141].

The plausible pathomechanisms attributed to myocarditis in long COVID patients is direct SARS-CoV-2 invasion of myocardial cells via ACE2 receptor, which resulted in local and systemic inflammation and led to myocardial damage, edema, and fibrosis. In parallel, ACE2 downregulation also increases the pro-inflammatory cytokines and inhibits anti-inflammatory cytokines, amplifying the inflammation process [14, 16].

#### 5.2.7 Arrhythmias

An observational study conducted by Zhou *et al*. [98] showed that the prevalence of specific arrhythmias, such as atrial fibrillation in long COVID is 1%. Nonetheless, no other research reported any other sort of arrhythmia. The low incidence of arrhythmia in the study is probably underreported due to transient arrhythmia cases. In contrast, an observational study showed that long COVID patients experienced palpitations ranging from 9% to 10.9% of patients [83, 142]. Thus, Holter monitoring is mandatory to diagnose transient arrhythmia [143].

Based on COVID-19 pathomechanisms, many types of arrhythmias could arise in long COVID. Firstly, the downregulation of ACE2 can lead to myocardial fibrosis, increased sympathetic stimulation, and atrial and ventricular potential action prolongation [54, 55]. Myocarditis due to direct SARS-CoV-2 infection can also disrupt the heart’s conduction system through the fibrosis process [15]. Taken together, all of the pathomechanisms converge to precipitate atrial tachycardia, atrial fibrillation, atrial flutter, ventricular tachycardia, or ventricular fibrillation.

## 6. Conclusions

The COVID-19 pandemic is an ongoing catastrophic public health event with dire long-term consequences, as many COVID-19 survivors experience a novel syndrome designated as long COVID syndrome. This novel syndrome also involved the CV system and manifests in coronary artery disease, hypertension, arrhythmia, heart failure, venous thromboembolism, and POTS. Thus, an approach is needed to achieve an early diagnosis, which enables the prevention of a severe disease’s course and improves the survivors’ quality of life as a whole. Nevertheless, further research on this novel syndrome, especially regarding its impact on CV, is warranted to fill in the research gaps.
